# A Novel Method of Closed Reduction and Percutaneous Pinning With Six K-wires for Intra-articular Fractures of the Distal Radius

**DOI:** 10.5435/JAAOSGlobal-D-19-00114

**Published:** 2020-06-01

**Authors:** Anilkumar Vidyadharan

**Affiliations:** From the Consultant Orthopedic Surgeon, Semalk Hospital, Ottapalam, India.

## Abstract

The existing method of closed reduction cannot reduce the complete intra-articular fracture of the distal radius; none of the available methods of percutaneous pinning alone can fix the intra-articular fracture of the distal radius. A prospective study including 108 complete intra-articular fractures of AO type B and C in 108 patients was conducted from 2013 to 2018. The subjects were aged between 18 and 84 years and included 61 men and 47 women. The cohort was divided into three age groups: ages 18 to 44, ages 45 to 59, and ages 60 to 84. The surgical technique includes an innovative method of closed reduction of the fracture in four steps, namely, distraction, compression, milking of the comminuted fragments, and repositioning of distal radio-ulnar joint, and a novel method of percutaneous pinning by creating two triangles in two planes by using six k-wires (1.6 mm) by encircling the metaphysis without interfering with the radio-carpal joint. Metaphyseal collapse is prevented by the role of k-wires such as the steel scaffolding in cement concreting, thereby maintaining the congruity of the radio-carpal joint and negative ulnar variance. Excellent and good results come to 87%. The patients can return to their original employment within 3 to 6 months in contrast to the other methods.

Fractures of the distal radius are the most common fractures of the upper extremity, of which 50% are intra-articular. The widespread use of radiographs, the direction and degree of displacement, and the presence or absence of articular injury lead to the development of various classification systems. This starts from Nissen-Lie in 1939 and Gartland and Werley in 1951 and later by various classifications such as Lindstrom, Older, Frykman, Melone, AO classification, Fernandez, Mayo clinic classification, fragment-specific classification, and columnar classification.^1^ Unlike the more common, lower-energy, extra-articular fractures, intra-articular fractures of the distal radius represent a complex injury that is associated with considerable morbidity. Generally, prognosis is less favorable for displaced, comminuted, intra-articular fractures. Longitudinal traction and manipulative reduction are generally ineffective because articular fragments often lack capsular or ligamentous attachments and thus do not respond to “ligamentotaxis” alone. More aggressive treatment regimens are generally required to anatomically restore the articular surface because of the inherent instability of the fracture and the tendency for articular fragments to settle after stress relaxation of the tensioned soft-tissue envelope. The conventional modes of treatments are closed reduction and casting (either plaster of paris or synthetic material), external fixation, or open reduction internal fixation.

During the past 2 decades, open reduction internal fixation techniques have been developed to address the comminuted intra-articular fracture of the distal radius, and in some cases, a combination of more than one surgical method is used such as external fixation, percutaneous pinning, and arthroscope-assisted surgeries.^2^ Still, complications are inevitable in one form or the other. Open techniques generally require extensive soft-tissue dissection and have been associated with higher complication rates than with the closed techniques. Wrist arthroscopy plays a good role in the treatment of intra-articular fractures.^3^ Limited incision approaches and low-profile modular implants have been introduced to reduce the complications. The treatment of severely comminuted and intra-articular fractures of the distal radius is still a challenge to the orthopaedic surgeons. In 1908, Lambotte described the placement of a single pin into the radial styloid to stabilize a displaced fracture of the distal radius.^1^ Later modifications of the technique by Stein and Katz in 1975, followed by Uhl, Lortat-Jacob, and Mortier in 1976 and intra-focal pinning by Kapandji in 1976 and 1986 are only applicable to extra-articular fractures. The role of percutaneous pinning in intra-articular fracture of the distal radius is only an add-on procedure to other modes of treatments such as arthroscope-assisted closed reduction and internal fixation, open reduction internal fixation, or external fixation. This is because of the belief that pinning is not a stable method of fixation against the deforming forces.

We present a novel technique of closed reduction and percutaneous pinning that alone can reduce and withstand the deforming forces in a complete intra-articular fracture of the distal radius. In addition to ligamentotaxis, this method of closed reduction is done by adding a milking technique to reposition the comminuted fragments and the fixation is performed by crossing six k-wires through the boundary of the distal radius essentially creating two triangles in two different planes, thus preventing a metaphyseal collapse. The two k-wires passing through an intact ulna act as a fulcrum to counter the deforming force emerged by the pull of brachioradialis muscle and thereby help to prevent the loss of radial tilt and maintain the length. The two transverse pins, with the help of two proximal pins directing toward the lunate facet, reduce and maintain the intra-articular fragments in position. We present a prospective study evaluating the results of this technique in 108 intra-articular fractures of the distal radius.

## Methods

A prospective study including 108 complete intra-articular fractures of AO type B and C in 108 patients was conducted from 2013 to 2018. The subjects were aged between 18 to 84 years and included 61 men and 47 women. Subjects who were not willing to undergo surgery were excluded. The cohort was divided into three age groups: ages 18 to 44, ages 45 to 59, and ages 60 to 84. Ninety percent of the cases were done within 2 to 24 hours after injury, and the maximum delay for surgery was 3 weeks. The duration of surgery was 30 to 45 minutes. Surgery was done under regional block or short general anesthesia. Patients were followed for 3 years.

### Closed Reduction

Distraction of the fracture is the first step, and it is done manually by the surgeon with a firm grip of the thumb of the patient with one hand and the index and middle fingers of the patient with the other hand while the counter-traction is done by the first assistant. Then, the hand is transferred by the surgeon to the second assistant in two stages. In the first stage, the index and middle fingers of the patient is transferred without loss of distraction and simultaneous shift of the surgeon's hand to the medial four metacarpals of the patient. In the second stage, the patient's thumb is transferred to the second assistant. Always use the medial three fingers of the second assistant in the supinated position in the extended elbow to hold the thumb of the patient so that the thumb of the patient will not slip and also gives maximum space for the surgeon to insert the Kirschner wire from the desired level of the radial styloid.

**Figure 1:**
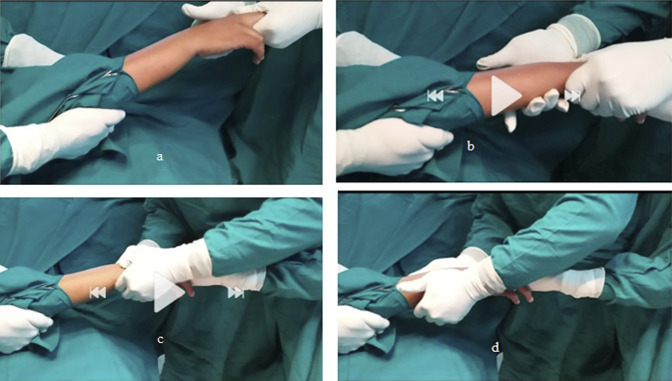
Photograph showing closed reduction. **A**, Distraction. **B**, Compression. **C**, Milking of the comminuted fragment. **D**, Repositioning of the distal radio ulnar joint.

AP compression of the fracture fragments is the second step and is done quickly by using both hands of the surgeon in the distracted position of the fracture fragments. Milking of the comminuted fragments is the third step. This is done with both thumbs and index fingers of the surgeon from the proximal fragment to the radiocarpal joint of the distal fragment. This will reduce the comminuted extra- and intra-articular fragments to the anatomic position. Finally, repositioning of the distal radioulnar joint is done by aligning the ulnar head about the sigmoid fossa of the radius with gentle compression (Figure 1).

### Percutaneous Pinning

Interfragmentary fixation is done by using six k-wires (size 1.6 mm) to create two triangles. The six k-wires are inserted percutaneously under the C-arm guidance, avoiding the radiocarpal joint and at the same time encircling all the comminuted metaphyseal fragments with the incorporation of the ulnar head and the distal radioulnar joint. On completion of the pinning, there should be at least two k-wires in each fracture fragment.

**Figure 2:**
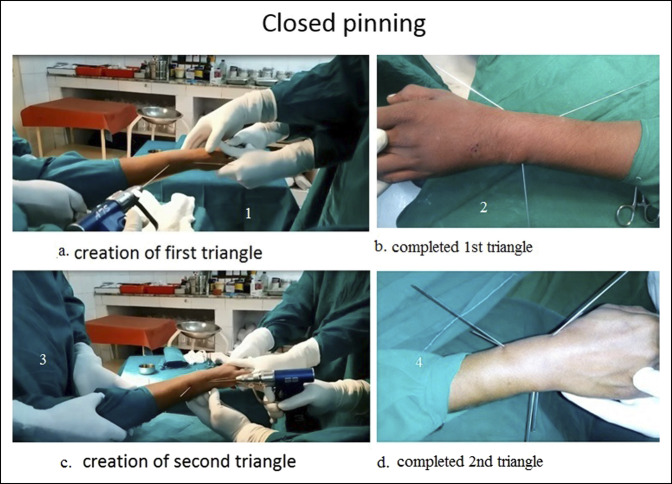
Photograph showing closed pinning. **A**, Creation of the first triangle. **B**, Completed the first triangle. **C**, Creation of the second triangle. **D**, Completed the second triangle.

Creation of the first triangle is done as quickly as possible, based on the clinical judgment of the reduction and preoperative planning of the pinning from the prereduction radiographs. The first Kirschner wire is introduced from the radial styloid to the medial side of the proximal fragment at an angle of 40° to 60° with the long axis of the forearm and 10° to 20° dorsally to the horizontal plane. The second Kirschner wire is introduced from the lateral side of the proximal fragment toward the radio-lunate facet (die punch) in the same plane as that of the first. The third Kirschner wire is introduced from the medial side of the ulnar head to the radial styloid through the distal radioulnar joint. Once the first triangle is completed, the triangular Kirschner wire frame will encircle the comminuted triangular metaphyseal area and will prevent the loss of the achieved reduction. The accuracy of the reduction and position of the k-wires are checked at this stage with the C-arm, and if any correction of reduction or pinning is required, that portion alone can be corrected without disturbing the other pins (Figure 2, A and B).

**Figure 3:**
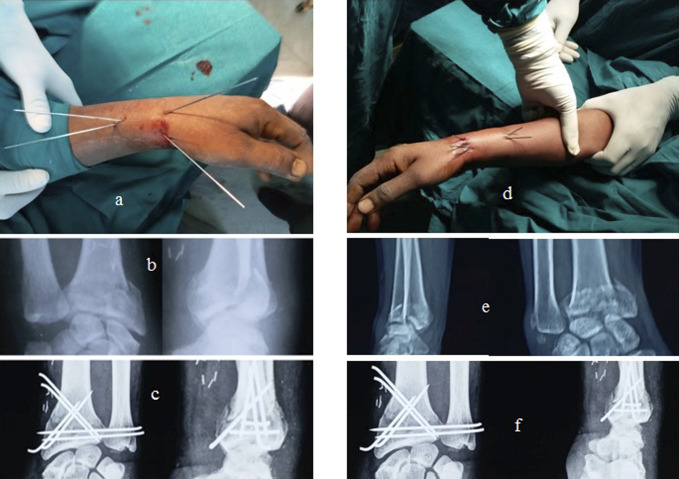
Photograph showing pinning of fractures with coronal fragments. **A**, Modification of the plane of the triangles. **B**, Showing the coronal fragments. **C**, Post-op radiograph showing the orientation of the k-wires. **D**, Additional k-wire set for engaging the coronal split. **E**, Pre-op radiograph showing the coronal split. **F**, Post-op radiograph showing the orientation of the k-wires.

If the reduction and primary fixation are satisfactory on imaging, three more k-wires are used for the creation of the second triangle in a different plane with the same orientation, with the help of the protruding ends of the first set of k-wires as a guide (Figure 2, C and D). Imaging is used to check the position and orientation of the second triangle. The ends of the k-wires are bend at 90° at the level of the subcutaneous tissue with the tip of a Kirschner wire bender by pressing the skin and subcutaneous tissue. The projecting end of the Kirschner wire is cut close to the bend so that it can be easily placed into the subcutaneous plane by pulling the overlying skin with a thumb forceps. A modified functional wrist splint which allows the complete range of finger movements and partial wrist movement was applied for 3 to 4 weeks. In severely comminuted unstable fractures, a volar plaster of paris slab in functional position is used instead of a splint. In coronal split fractures, the selection of the plane of the triangles and direction of the k-wires are modified to include the comminuted fragments and if necessary, additional k-wires are used (Figure 3).

### Postoperative Protocol

**Figure 4:**
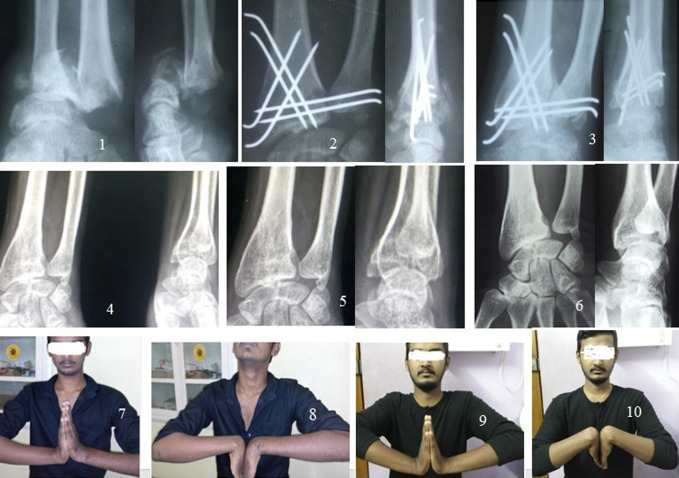
Photograph showing a 21-year-old man from day 1 to 3 years (left wrist). **A**, Pre-op radiograph. **B**, Post-op radiograph. **C**, radiograph at 6 weeks. **D**, radiograph at 3 months. **E**, radiograph at 6 months. **F**, radiograph at 3 years. **G**, Dorsiflexion at 3 months. **H**, Palmar flexion at 3 months. **I**, Dorsiflexion at 3 years. **J**, Palmar flexion at 3 years.

**Figure 5:**
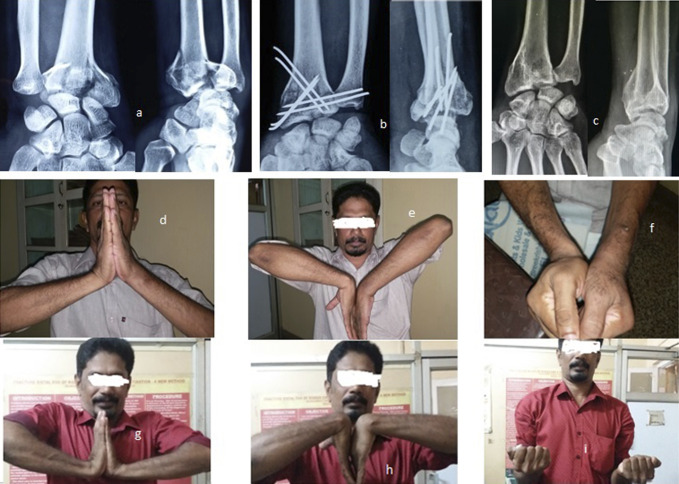
Photograph showing a 46-year-old man from day 1 to 1 year (left wrist). **A**, Pre-op radiograph. **B**, Post-op radiograph. **C**, radiograph at 1 year. **D**, Dorsiflexion at 6 weeks. **E**, Palmar flexion at 6 weeks. **F**, Grip at 6 weeks. **G**, Dorsiflexion at 1 year. **H**, Palmar flexion at 1 year. **I**, Grip and supination.

Patients are discharged on the second day with a functional splint or plaster of paris slab with active finger movements and partial wrist movement and are advised to do some light personal activities such as writing, buttoning, shaving etc. Patients are reviewed at the end of the first week to check their range of motion and on the third week to check radiograph. The splint is removed after 3 to 4 weeks to allow enhanced wrist exercise programs for another 2 to 3 weeks, and the k-wires are removed after 6 weeks. The patients were followed up at 2 months, 3 months, 6 months, 1 year, 2 year, and 3 year (Figures 4 and 5).

The subjects were evaluated based on the clinical scoring system of Green and O'Brien modified by Cooney based on four criteria, namely, pain, functional status, grip strength, and the range of movement (Table 1). The wrist questionnaire was completed by the patients at 2 months, 3 months, 6 months, and 1 year (Tables 2, 3, and 4).

**Table 1:** Clinical Scoring System of Green and O'Brien, modified by Cooney

Criteria	Findings and Score	Findings and Score	Findings and Score	Findings and Score	Findings and Score
Pain	25	20	15	0	
	No pain	Mild pain	Moderate	Severe	
Functional Status	25	20	15	0	
	Returned to regular employment	Restricted employment	Restricted but unemployed	Unable to work	
Grip Strength	25	15	10	5	0
	Normal	75%-99% of normal	50%-74% of normal	25%-49% of normal	0-24% of normal
Range of movement	25 (normal)	15 (75%-99% of normal)	10 (50%-74% of normal)	5 (25%-49% of normal)	0 (0-24% of normal)
	Palmar flexion dorsiflexion arc is more than 120°	Palmar flexion dorsiflexion arc is 91°-119°	Palmar flexion dorsiflexion arc is 61°-90°	Palmar flexion dorsiflexion arc is 31°-60°	Palmar flexion dorsiflexion arc is less than 30°
Final score	Excellent	Good	Satisfactory	Poor	
	90-100	80-89	65-79	Below 65	

**Table 2:** Wrist Questionnaire _______________ Patient name: _______________Serial. No: _______________ Review Period: _______________

Wrist Questionnaire	Preinjury			_______________ After Surgery			
General Activities	Yes	No	NA	HT	Yes	No	NA
Washing the body							
Washing hair							
Combing and styling hair							
Putting on underwear							
Pulling up trousers							
Tying shoelaces							
Using eating utensils							
Pulling up a zipper							
Fastening buttons							
Handling money							
Undoing a screw-top lid							
Opening a door							
Turning on a tap							
Accessing a vehicle							
Pulling an electrical plug out of a socket							
Washing dishes							
Lifting a saucepan							
Sweeping							
Gardening							
Dusting or polishing							
Lifting activities							
Craft activities							
Caring or playing with children							
Steering the car							
Taking weight through the wrist							

NA = not applicable, HT = haven't tried.

**Table 3:** Patient Name: _______________ Serial. No: _______________ Review Period: _______________

Wrist Questionnaire-Pain Status	Pre injury			_______________ After Surgery			
	Yes	No	NA	No Pain	Mild Pain	Moderate Pain	Severe Pain
Work							
Feeding							
Intimate moments							
Cooking							
Hygiene activities							
Sport/hobbies							
Washing							
Transport							
Outside duties							
Cleaning							
Sleep							
Dressing							
Caring for family							

NA = not applicable.

**Table 4:** Patient Name: _______________ Serial. No: _______________ Review Period: _______________

Wrist Questionnaire-Functional Status	Pre injury			_______________ After Surgery			
	Yes	No	NA	Return to Regular Work	Return to Restricted Work	Restricted-Unemployed	Unable to Work
Work							
Feeding							
Intimate moments							
Cooking							
Hygiene activities							
Sport/hobbies							
Washing							
Transport							
Outside duties							
Cleaning							
Sleep							
Dressing							
Caring for family							

NA = not applicable.

## Results

On analysis of the results, within the first 3 months, 75% of patients had either no pain or only mild pain and 70% of patients regained above 75% of range of movement. Within 3 months, 50% of patients returned to either regular or restricted employment and had more than 50% of their contralateral grip strength (Table 6).

**Figure 6:**
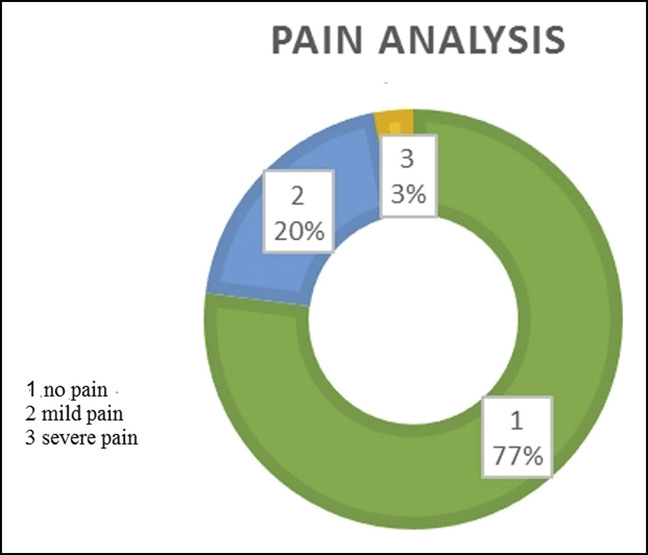
Illustration showing a detailed analysis of the criterion “pain.”

**Figure 7:**
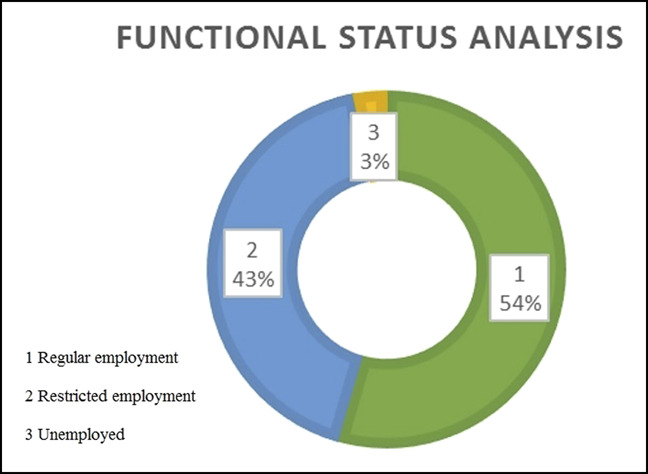
Illustration showing a detailed analysis of the criterion “functional status.”

**Figure 8:**
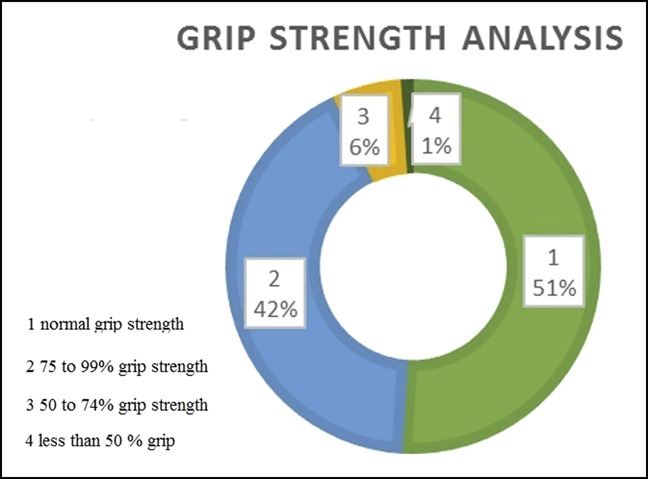
Illustration showing a detailed analysis of the criterion “grip strength.”

**Figure 9:**
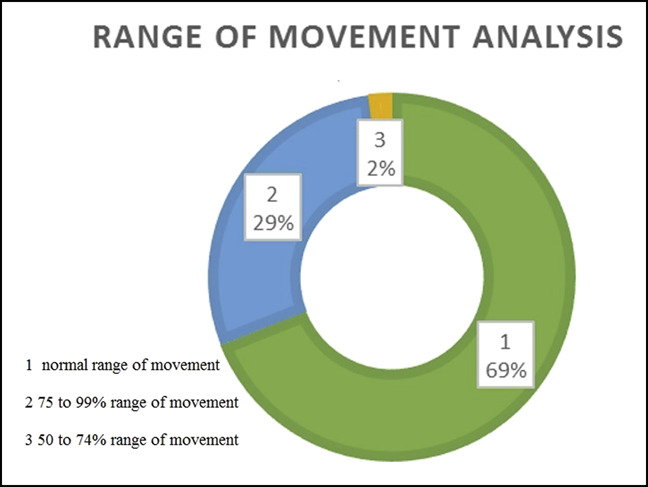
Illustration showing a detailed analysis of the criterion “range movement.”

At the end of 6 months, 97% of patients had either no pain or only mild pain (Figure 6). Within 6 months, 97% of cases returned to their original employment, either in the regular or restricted form (Figure 7). At the end of 6 months, 93% of patients regained either full or more than 75% of the grip strength (Figure 8). Within 6 months, 98% of patients regained either full or more than 75% of the normal range of movement (Figure 9).

By the end of the first year, only 3% cohorts had mild pain and there were no reports of moderate or severe pain. By the end of 1 year, 88% of patients regained the normal grip strength and 98% of patients regained the normal range of movement.

The analysis of the results revealed that painless wrist activities were possible from the immediate postoperative period, and 97% of patients returned to their original work within 3 to 6 months (Table 5). The outcome of questionnaire and measurements were summarized (Table 6).

**Table 5:** The Summary of the Study Results of the Innovative Surgery at 6 Months

Group	Total Number	Excellent	Good	Satisfactory	Poor	Sum of Excellent and Good	Percentage
Group-I	47	25	18	3	1	43	91
Group-II	34	16	13	4	1	29	85
Group-III	27	12	10	4	1	22	81
Total all	108	53	41	11	3	94	87

**Table 6:** Outcome of Questionnaire and Measurements (Total Number of Cases: 108)

Criteria	2 mo	3 mo	6 mo	12 mo
Pain				
No pain	24	55	83	105
Mild pain	25	33	22	2
Moderate pain	46	17	3	1
Severe pain	13	3	0	0
Functional status				
Returned to regular employment	0	45	59	95
Restricted employment	44	43	46	11
Restricted but unemployed	46	17	3	2
Unable to work	18	3	0	0
Grip strength				
Normal	0	30	55	95
75%-99% of normal	5	42	45	7
50%-74% of normal	32	17	7	5
25%-49% of normal	59	11	1	1
0-24% of normal	12	8	0	0
Range of movement				
Normal	5	30	75	105
75%-99% of normal	39	45	31	3
50%-74% of normal	46	23	2	0
25%-49% of normal	13	7	0	0
0-24% of normal	5	3	0	0

mo = months.

The radiological analysis revealed that radial length, radial inclination, palmar tilt, ulnar variance, and articular congruity were maintained to the initial postoperative alignment using this novel pinning method (Table 7).

**Table 7:** Radiological analysis

Time Interval	Radial Length	Radial Inclination	Volar Tilt	Ulnar Variance
Preoperative, mean value	5.45	12.88	−19.3	3.50
Immediate postoperative, mean value	12.11	22.58	10.30	−1.67
2 mo after surgery, mean value	11.87	22.31	10.10	−1.47
3 mo after surgery, mean value	10.32	21.83	9.97	−1.30
6 mo after surgery, mean value	10.20	21.65	9.73	−1.25
1 yr after surgery, mean value	10.13	21.52	9.60	−1.20
2 yr after surgery, mean value	10.13	21.52	9.60	−1.20
3 yr after surgery, mean value	10.13	21.52	9.60	−1.20

Time Interval	With intra-articular Step		Intra-articular Gap
	<2 mm	>2 mm	No Cases
Preoperative	64	44	46
Immediate postoperative period, number of patients	14	0	0
2 mo after surgery, number of patients	14	0	0
3 mo after surgery, number of patients	8	2	3
6 mo after surgery, number of patients	4	2	3
1 yr after surgery, number of patients	3	2	3
2 yr after surgery, number of patients	3	2	3
3 yr after surgery, number of patients	3	2	3

## Complications

**Figure 10:**
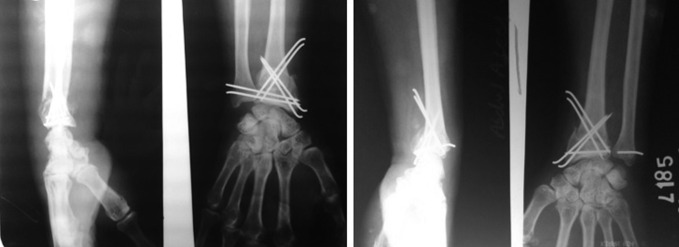
Radiograph showing pin breaking.

Pin loosening and pin migration happened in severely osteoporotic bones with overexercise program. Pin breaking occurred in two cases in the transverse pin because of excessive rotational force. Both the broken pins were removed by pushing with tight cannulated drill bits through the proximal hole to the opposite side. No deep infection was noticed in any case, but a superficial skin infection was observed in pin migrated cases. Residual deformity was very minimal. CRPS was very low, and all of them were type 1 (Figure 10 and Table 8).

**Table 8:** The Details of the Complications of This Surgical Procedure

Group	Pin Migration	Pin Loosening	Residual Deformity	Skin Necrosis	Deep Infection	Pin Breaking	CRPS- (Only Type-1)	Osteoarthritis (Only Radiological Evidence)
Group-I	1	1	1	2	0	1	1	1
Group-II	1	3	2	3	0	1	2	2
Group-III	2	4	3	4	0	0	3	2
Total	4	8	6	9	0	2	5	5

CRPS = complex regional pain syndrome.

## Summary

In the treatment of an intra-articular fracture of the distal radius, at present, closed reduction treatment is advocated only for the AO type A fracture. AO type B and C fractures are treated by surgical methods such as open reduction or arthroscope-assisted closed/open reduction.^3^ The conventional pinning is not a stable fixation to withstand the deforming forces acting during the fracture healing, and hence, it is used as an add-on procedure to the other surgical methods. However, in this new method of pinning, stability is achieved by the crossing of the six k-wires at six points with at least two k-wires in each interfragmentary fixation and thereby preventing pin migration and pin loosening.

On the analysis of the findings of this surgical procedure, the advantages can be summarized as follows.

(1) Early return to original employment is possible between 3 and 6 months because of the early return of the range of movement of the joints and restoration of the grip strength, which is not possible in other surgical methods
(2) Intra-articular fractures can be reduced by a modification of the existing closed reduction technique by adding a milking technique in the completely distracted position. In the conventional open reduction internal fixation treatment of the intra-articular fractures, there will be further soft-tissue damage. This will result in permanent joint stiffness and in turn reduces the grip strength and delay in return to the original employment.^2^
(3) Because there is no skin incision and no implants projecting outside the skin, painless movement of the fingers and radiocarpal joint is possible from the immediate postoperative period. Owing to the stability of the fixation, the patient can do some form of personal work from the immediate postoperative period, which in turn avoids the usual joint stiffness, thereby reducing the chance of complex regional pain syndrome (CRPS) in contrast to the other surgical methods such external or internal fixations.^4^ In this study, only five patients sustained type-1 CRPS with mild pain and a restriction of joint movements. All of them recovered within 3 to 6 months with active exercise and NSAID.
(4) Pin loosening, pin migration, skin necrosis, metaphyseal collapse, residual deformity, and CRPS are common complications in conventional pinning, and hence, radial shortening, loss of palmar tilt, and incongruity of the articular surface are common.^5^ However, in this method of pinning, metaphyseal collapse is prevented by the role of six k-wires in a crossed manner to form two closed triangles through the triangular-shaped outer boarders of the distal radius such as the role of steel scaffolding in cement concreting. In this study, there were pin migration in four cases where the end of the Kirschner wire had overpenetrated in the opposite cortex and three of four were osteoporotic bone. Because of the engagement of at least two pins in each interfragmentary piece in each direction, the loosening of one of these pins will not affect the strength or stability of the fixation. This can be avoided by engagement of the tip of the Kirschner wire in the opposite cortex or by using k-wires having threaded tips. In patients with less bone mass or more comminution, a plaster of paris slab is applied instead of splint for the first 4 weeks. Eight patients sustained loosening of both pins of the same direction. Unlike the conventional pinning technique, no deep infection or severe skin necrosis was present. This is because there is no skin incision and no implants projecting outside the skin. In this study, there were only superficial skin necrosis in nine patients because of pin loosening and were healed after Kirschner wire removal with oral antibiotics.
(5) Here, the congruity of the radiocarpal joint is maintained by preventing the stepping due to the metaphyseal collapse with the help of the two transverse pins parallel to the articular surface and the two proximal pins directed toward the radio-lunate facet (die-punch). Five patients sustained radiological evidence of osteoarthritis without many clinical symptoms. They were only followed for a short term and will likely develop posttraumatic osteoarthritis.
(6) In other surgical methods except arthroscope-assisted reduction and fixations, the reduction and maintenance of a disrupted distal radioulnar joint is a difficult task and damages of the soft-tissue structures because of open procedures and may result in joint stiffness.^1^ However, here, the two transverse pins passing through both the cortices of an intact ulna act as the fulcrum for countering the rotational forces and pull of brachioradialis and hence stabilizing the distal radioulnar joint and thereby preventing the radial inclination deformity, radial shortening, and helps to maintain the negative ulnar variance.
(7) Pin breaking of the transverse pins can happen because of the forced rotation of the wrist, especially in pin migration cases. This can be avoided by engaging the two transverse pins in both the cortices of the ulna and radius. In pin loosened and pin migrated cases we can extend the pop slab until Kirschner wire removal.

In severely comminuted intra-articular fractures or osteoporotic fractures, there are chances for pin migration and pin loosening, and it can be avoided by applying a volar plaster of paris slab in the functional position of the wrist, instead of splint. The chances of malunion are very low because of the prevention of the metaphyseal collapse. The occurrence of CRPS is eliminated or reduced to a minimum by the painless early active joint movements of the wrist and fingers. The joint stiffness is less in innovative pinning. There is no tendon or nerve injury in this method because the site of penetration of the pins can be selected by avoiding tendon and nerves.^2^

Recent literature shows that specific technique is not as important as attaining anatomic reduction. The ability of the surgeon to restore anatomy with the least invasive procedure results in the quickest functional return.^3^

## References

[1] Slutsky D: Fractures and Injuries of the Distal Radius and Carpus: The Cutting Edge*.* Philadelphia, Saunders Elsevier, 2009, pp 4-9.

[2] Canale T Beaty JH: Campbell's Operative Orthopaedics. ed 12*.* Philadelphia, Mosby, Elsevier, 2013, pp 2890-2894.

[3] Mehta JA Bain GI Heptinstall RJ: Anatomical reduction of intra-articular fractures of the distal radius: An arthroscopically assisted approach. J Bone Joint Surg Br 2000;82-B:79-86. 10.1302/0301-620x.82b1.10101 10697319

[4] Kreder HJ Hanel DP Agel J: Indirect reduction and percutaneous fixation versus open reduction and internal fixation for displaced intraarticular fractures of the distal radius: A randomized, controlled trial. J Bone Joint Surg Br 2005;87-B:829-836. 10.1302/0301-620X.87B6.15539 15911668

[5] Handoll HHG Vaghela MV Madhok R: Percutaneous pinning for treating distal radial fractures in adults (review). Cochrane Database Syst Rev 2008;4:4-6. 10.1002/14651858.CD006080.pub2 17636827

